# Plant biotechnology research at forest fields in South Korea

**DOI:** 10.1186/1753-6561-5-S7-P184

**Published:** 2011-09-13

**Authors:** Jae In Park Chungbuk

**Affiliations:** 1 National University, Korea

## 

This paper is to give an outline of the tree biotechnology research in South Korea.

Tree Breeding, we call Traditional Tree Breeding later compared to Tree Biotechnology, started in 1954 in South Korea. Tree Biotechnology Research started as Tissue culture in the late 1970 in South Korea. Micropropagation was the first field adopted. Embryo culture, Bud culture, Callus culture, Cell culture are the methods for Micropropagation. Many tree species and herbaceous plants have been subjected to micropropagation and successfully established. Tall tree species, *Populus alba* ×*P. glandulosa*, *Quercus*, *Betula et al* are successful for plantation, rare woody plants such as *Forsythia saxatilis*, *Abeliophylum disticum*, *Berchemia berhemiaefolia*, *Hovenia dulsis*, *Lagerstroemia indica* for. *alba*, *Empetrum nigrum* var. *japonicum were* also subjected to in vitro culture*.* For somatic embryogenesis many species were successful in *Laxix leptolepis*, *Liriodendron tulipifera*, *Aralia elata*, and *Schisandra chinensis* et al*.* For Practical purpose a few species such as Mountain ginseng(root inductio from callus culture and subsequent root culture by liquid culture) , thornless *Aralia elata*(somatic embryogenesis form petioles), *Liriodendron tulipifera*(somatic embryogenesis from immature zygotic embryo) are under way of commercialization. Mountain ginseng is wild ginseng grown in mountain area. In mountain area they live very long time , even as long as over 100 years meanwhile the plants of it can grow only up to 6 years and produce healthy secondary products for human body. After Multiplication the produced roots are utilized for liquor, cosmetics, medicines *et al*. Yellow poplar**(***Liriodendron tulipifera***),** introduced from north America**,** shows good growth performance and is planted widely throughout the country. Through mass propagation of superior trees by somatic embryogenesis clonal forestry is planned in the forest area.

Biosafety policy of south Korea is very strict. Cultivation of transformed plants is not allowed.

The following list shows the outline of the species which have been reported to be successful to the category in south Korea

**Bud culture;***Populus alba* ×*P. glandulosa***,***Kalopanax septemlobus*, *Corylopsis coreana*, *Eucalyptus pellita*, *Actinidia deliciosa* × *A. arguta*, *Prunus yedoensis*,*Diospyros kaki . Phellodendron amurese*, *Robinia pseudoacacia*, *Quercus acutissima .*

Forsythia koreana for. aureoreticulata, Salix hallasanensis, Sorbus commixta, Betula schmitti, Machilus thunbrgii, Aconitium koreanum, Juglans regia, Betula dahurica, Crataegus pinnatifida,

**Callus cuture;***Pinus koraiensis*, *Ailanthus altissima*, *Medicago sativa*, *Lycium chinense*, *Robinia pseudoacacia* wild Panax ginseng**, Populus koreana × P. nigra var. italica,**

**Cotyledon culture;***Pinus rigida* × *P. taeda*

**Cell culture;***Salix koreensis*, *Gardenia jasminoides*, *Taxus cuspidata*, *Hibiscus syriacus*,

**Embryo culture;***Pinus rigida*× *P. taeda***,***Camelia sinensis*, *Pimpinella brachycarpa*, *Populus glandulosa*,

**Leaf culture;***Camelia sinensis*

**Cambial tissue culture***; Larix decidua*

**Somatic embryogenesis ;***Populus nigra*× *P. maximowiczii*, *Zizyphus jujuba*, *Oplopanax elatus***,***Eleutherococcus koreanum*, *Kalopanax pictus*, *Aralia elata”Zaoh”*, *Liriodendron tulipifera*, *Larix kaempferi*, , *Lycium chinense*, *Camelia sinensis*, *Orostachys japonicus*, *Quercus variabilis*, *Pimpinella brachycarpa*, *Juglans regia*

**Anther culture*;****Populus glandulosa*

**Protoplast isolation or culture;***Populus alba* ×*P. glandulosa*, *Populus euramericana Populus alba*, *Populus glandulosa*, *Populus nigra*×*P. maximowiczii*

**Bioreactor culture;***Ganoderma applanatum***, **, *Eleutherococcus koreanum*, *Tricholoma matsutake*(mycelium), *Lilium ‘Casa Blanca’*, *Acanthopanax semticosus***, ***Acanthopanax koreanum***, *wild Panax ginseng***

**Molecular markers such as I-SSR marker, cpSSR marker have been developed in***Quercus acutissima*, *Pinus densiflora*, *Salix koreensis***for forensic medicine**

**Functional genomics:***Populus alba* ×*P. glandulosa*, *Magnolia kobus*

**Quantitative Traits Loci for root growth**; *Populus davidiana*

**Root culture***; Albizzia kalkora*, *Eleutherococcus koreanum*, *Tripterospermum japonicum*

**Transformation***: Populus alba***×***P. glandulosa*(Model tree), *Aralia elata*, *Populus koreana* × *P. nigra*, *Camellia chinensis*, *Populus nigra*, *Populus davidiana*, *Pinus densiflora*, *Quercus acutissima*,

**I-SSR analysis***; Stewartia koreana*, Genus*Juglans*, *Vaccinium uliginosum*, *Oplopanax elatus*,*Eleutherococcus senticosus*, *Ginkgo biloba*, *Abies holophylla'*

*Torreya nucifera*, *Ginko biloba*, *Rhododendron schlippenbachii*,*Pinus densiflora*, *Taxus cuspidate*, *Thuja koraiensis*

**Cryopreservation***; Bursaphelenchus xylophilus*(*Pine Wood Namatode*), ***Populus koreana* × *P. nigra var. italica***, *Populus alba***× ***P. glandulosa*

**Expressed Sequence Tags***; Populus alba* × *P. tremula var. glandulosa*

**Microsatellite markers***; Quercus acutissima*

**Secondary Metabolites***: Eleutherococcus chiisanensis*(*eleutherosides*), *Cornus walteri*

*Amorpha fruticosa*, *Eleutherococcus senticosus*, *Piper nigrum*, *Kalopanax pictus*, *Grifola umbellate*(Sclerotium)*Populus alba* ×*P. glandulosa*(anthocyanin), Taxus cuspidate, *Phellodendron amurense*, *Camelia chinensis*

**Organogenesis**; Orchard grass

**Phytoremediation**; *Populus alba***×***P. glandulosa*, *Populus nigra***×***P****.****maximowiczii. Populus euramericana*

**Allelopathy;***Phellodendron amurense*,

**Cryopreservation;***Sapindus mukorossi*, *Lycium chinense*, *Melia azedarach*

Acer mono

